# Psychiatric morbidity in non-psychiatric geriatric inpatients

**DOI:** 10.4103/0019-5545.31621

**Published:** 2006

**Authors:** Aman Sood, Paramjit Singh, Parshotam D. Gargi

**Affiliations:** * Junior Resident, Department of Psychiatry, Medical College, Amritsar, punjab; ** Professor and Head, Department of Psychiatry, Medical College, Amritsar, punjab; *** Associate Professor, Department of Psychiatry, Medical College, Amritsar, punjab

**Keywords:** Geriatric, psychiatric morbidity, liaison psychiatry

## Abstract

**Aim:**

To evaluate the profile of psychiatric disorders in geriatric inpatients.

**Methods:**

A total of 528 patients (age 65 years and above) admitted to various departments of the teaching hospital attached to the Government Medical College, Amritsar from 15 September 2001 to 14 September 2002 were included in the study. Psychiatric assessment of patients was made on the basis of psychogeriatric assessment scales (PAS) and present state examination (PSE-ninth edition, 1974). The ICD-10 criteria were used for psychiatric diagnoses. General medical conditions were diagnosed by consultants of the respective departments. The patients were finally assessed by the consultant of the Department of Psychiatry. The obtained data were analysed using the chi-square test.

**Results:**

Of the 528 patients, 260 (49%) had psychiatric co-morbidity. The most common psychiatric disorder was depression (25.94%), followed by adjustment disorders (11%), anxiety disorders (4.54%), dementias (3.6%), delirium (3%), bipolar disorders (0.8%), and substance-related disorders (0.4%).

**Conclusion:**

The above findings emphasize the importance of consultation-liaison psychiatry, especially in geriatric patients.

## INTRODUCTION

Ageing is a universal phenomenon. It has not only social but also economical, political and health-related implications. This ever-increasing age group needs special healthcare. Psychological assessment should be an integral aspect of the comprehensive functional health assessment of geriatric patients. In recent years, problems related to old age are getting recognition. The United Nations General Assembly resolved to observe 1999 as the International Year of Older People. The theme for the World Health Day on 7 April 1999 was ‘Active ageing makes the difference’.

Various studies have evaluated psychiatric disorders in elderly medical–surgical patients and have found a significant positive role of these disorders in the final outcome. In the 1970s studies were conducted in India to know the psychiatric profile of the geriatric population.^1^–^5^ Schuckit *et al.*^6^ observed at La Jolla Veterans Administration Hospital that 24% of medical–surgical patients over the age of 65 years had unrecognized major mental disorders, predominantly depression or alcoholism. Research diagnostic criteria were used in a structured interview to evaluate the presence of unrecognized psychiatric disorders in 50 acute medical and surgical patients. Field surveys of the geriatric population have also been attempted.^7^–^11^

Nandi *et al.*^10^ did a study in two villages of West Bengal with the aim of assessing mental morbidity of the elderly population aged 60 years and above. It was found that 61% of these elderly people needed psychiatric treatment. The overwhelming majority of affected persons were depressive. The prevalence of dementia was found to be 1.6%. Women had a higher rate of morbidity than men, i.e. 77.6% and 42.4%, respectively. Mehta *et al.,*^12^ Premarajan^13^ and Shaji *et al.*^9^ in their respective community-based studies on the elderly observed that more women suffered from psychiatric disorders as compared to men.

Tiwari and Srivastava^11^ conducted a study in Lucknow to assess the prevalence of psychiatric illness in persons 60 years and above in a defined rural community, and compared it with that of non-geriatric population to know the difference between the geriatric and non-geriatric segments. It was observed that psychiatric morbidity was much higher in the geriatric population (42.21%) as compared to the non-geriatric population (3.97%). The total prevalence of psychiatric disorders in the geriatric group was 42.21% and neurotic depression, MDP depressed and anxiety states were the most prevalent.

Avasthi *et al.*^14^ analysed retrospectively 1245 referrals seen over 7 years at PGIMER, Chandigarh. This study showed that psychiatric profiles in referrals from different subpopulations divided according to age, gender, source of referrals and medical–surgical diagnosis were quite dissimilar. Out of these, 8.6% (107 cases) were in the geriatric age group (60 years and above). The frequency of distribution of psychiatric diagnoses among geriatric patients was 48 cases of organic psychoses, 26 neurosis, 19 other disorders and 14 had no psychiatric disorders.

Soo and Unutzer^15^ reported that the relation between functional health and psychiatric co-morbidity has been clearly established for depression, and emerging data suggest similar trends for anxiety disorders, sleep disorders and psychosis. Medical co-morbidity aggregates with ageing, 40% of men and over 50% of women who live beyond the age of 70 years have two or more chronic disorders and most of those over 80 years have multiple health problems requiring professional care. Muller *et al.*^16^ reported on psychiatric morbidity among elderly inpatients in Germany and found dementia to be the commonest disorder in 26%, followed by depressive disorders in 22%, alcohol abuse in 11%, and benzodiazepine abuse in 2%. Bowler *et al.*^17^ also reported that the prevalence of dementia in elderly inpatients was very high. They carried out their study in an acute inpatient medicine unit for the elderly; 153 patients were screened and interviewed in a two-stage diagnostic procedure. They observed that 11.1% of the study partici-pants were delirious, 26.8% were demented and 9.2% were depressed. In India, however, the prevalence of dementia as such is low as reported by earlier field surveys. Rao and Madhavan^8^ carried out their study in a semi-urban area near Madurai and found dementia in 6 cases out of 686 persons (0.88%). Ramachandran and Menon^2^ in their study reported a rate of dementia of 3.2%. Shaji *et al.*^18^ made a door-to-door survey of a rural area in Kerala and found that 3.39% of the population (aged 60 years and above) had dementia. White^19^ reviewed cross-cultural research on the epidemiology of dementia and came to the conclusion that the estimated rates of dementia were somewhat lower in Asian countries as compared with Europe and the USA.

Uwakwe^20^ evaluated psychiatric morbidity in elderly patients admitted to non-psychiatric wards in a general/teaching hospital in Nigeria. All patients aged 60 years and above who were admitted into medical, surgical and gynaecological wards were assessed with a self-reporting questionnaire, mini mental state examination and geriatric mental state schedule. Diagnoses of mental disorders were made according to the ICD-10 diagnostic criteria. It was found that mental morbidity was 45.3% with depression being commonest, followed by organic disorders (delirium, dementia), adjustment disorder and generalized anxiety disorder. There were also cases of alcohol and drug abuse. The physicians could recognize only 2.8% of mental disorders. In India, little attention has been paid so far to the psychiatric aspect of geriatric patients by medical and surgical specialists. This study was undertaken to draw attention to this aspect.

## METHODS

Patients 65 years of age or above admitted in various departments of the teaching hospital attached to the Government Medical College, Amritsar from 15 September 2001 to 14 September 2002 were interviewed. Of the 578 patients screened, 528 were included in this study. The remaining patients had to be excluded as 8 of them were unconscious, 36 were terminally ill and unable to communicate, 6 of them were excluded as no reliable informant was available.

### Inclusion criteria

Inclusion criteria of subjects for the study were:

- Age (65 years or above)
- Informed consent of the patient and his/her relatives to participate in the study taken

### Exclusion criteria

- Unconscious patient
- Uncooperative patient

Psychiatric assessment of patients was made on the basis of the psychogeriatric assessment scale and present state examination. Psychiatric diagnoses were made as per the ICD-10 criteria. General medical conditions were diagnosed by consultants of the respective departments. The cases were reviewed for final assessment by the consultant of the psychiatry department. The data were statistically analysed.

### 1. Psychogeriatric assessment scale

The psychogeriatric assessment scale (PAS) is a standardized interview for dementia, depression and related disorders in the geriatric population. A limitation of diagnostic criteria such as ICD-10 or DSM-IV is that they treat a syndrome as a categorical state, whereas there are good arguments that disorders such as dementia and depression form a continuum in the population.^21^,^22^ Mental state can be assessed in a continuous manner using scales of depressive symptoms such as the geriatric depressive scale^23^ and scales of cognitive impairment such as the mini mental state examination.^24^ However, these scales generally cover only a single aspect of the mental state and rely on only one source of information.

Jorm *et al.* (1993)^25^ developed PAS to gather information on major psychogeriatric disorders. The purpose of psycho-geriatric assessment scales is to provide a brief but comprehensive profile of an elderly person's mental state using a straightforward interview. The PAS was developed using CIE data from an epidemiological survey of the elderly in Canberra.^26^ The validity and reliability of the instrument was further assessed using CIE data from clinical samples in Sydney^27^,^28^ and Geneva.^29^

### 2. Present state examination

The present state examination (PSE) was developed by Wing *et al.*^30^ for current psychopathology, if any. PSE contains 140 items covering all topics of mental state such as worrying anxiety, concentration, mood affect, appetite, sleep, libido, obsessions–compulsions, perceptual disorders, thought influence and other delusions, sensorium, appearance, behaviour, speech and insight. It also allows the examiner to rate the adequacy of the interview and to quote the reasons for inadequacy, if any. PSE was used as a structured interview schedule that focused on symptoms that had occurred during the past month. Although PSE itself does not generate a diagnosis, it provides information that can be related to disease categories in the international classification of diseases (ICD).

## RESULTS

Of the 528 patients aged 65 years or above (mean age 70.5 years), 64% were men and 36% were women. The mean age of male inpatients was 68.80 years and for female inpatients 70.74 years.

The age distribution was as follows: 43.4% (229 cases: 129 males, 100 females) were in the age group of 65–69 years, 35.2% (186 cases: 124 males and 64 females) were in the age group of 70–74 years, 10.2% (54 cases: 40 males and 14 females) were in the age group of 75–79 years and 11.2% (59 cases: 46 males and 13 females) were in the age group of 80 years and above.

A total of 260 patients (49.28%) had psychiatric co-morbidity. The most common psychiatric illness (25.94%) was depression followed by adjustment disorders (11%), anxiety disorders (4.54%), dementias (3.6%), delirium (3%) bipolar disorders (0.8%), and substance-related disorders (0.4%).

Table 1 shows that the association between age and distribution of psychiatric disorders was not statistically significant, which indicates that psychiatric disorders did not vary much in the geriatric age groups; however, there was significant association between gender and psychiatric disorders.

**Table 1:** Age- and sex-wise distribution of patients with psychiatric co-morbidity (*n*=528)

	Psychiatric disorders								
		
Age groups* (years)	No psychiatric disorder	Depressive disorder	Adjustment disorder	Anxiety disorder	Dementia	Delirium	Bipolar disorder	Substance-related disorders	Chi-squareand probability
65-69 *n*=229	119 (52)	56 (24.45)	25 (10.9)	12 (5.24)	6 (2.62)	8 (3.5)	2 (0.8)	1 (0.4)	χ^2^=43.59 p< 0.01; df=7
70-74 *n*=186	92 (49.46)	57 (30.64)	20 (10.75)	7 (3.76)	4 (2.15)	4 (2.15)	2 (1.1)	0	χ^2^=49.78 p<0.01; df=7
75-79 *n*=54	27 (50)	12 (22.2)	7 (13)	2 (3.71)	4 (7.4)	1 (1.8)	0	1 (1.8)	χ^2^=38.93 p<0.01; df = 7
>80 *n*=59	29 (49.15)	13 (22.03)	6 (10.16)	3 (5.08)	5 (8.30)	3 (5.08)	0	0	χ^2^=34.82 p<0.01; df=7
Gender**									
Male *n*=339	186 (54.86)	75 (22.12)	35 (10.32)	13 (3.83)	15 (4.42)	9 (2.65)	4 (1.17)	2 (0.6)	χ^2^=46.59 p<0.01; df=7
Female *n*=189	82 (43.38)	62 (32.80)	23 (12.16)	11 (5.82)	4 (2.11)	7 (3.70)	0	0	χ^2^=44.98 p<0.01; df=7

Note: Values within parentheses are percentages df: degree of freedom

* Chi-square=17.63, df=21; p>0.05 (p=0.6725) (NS)

** Chi-square=15.50; df=7; p<0.05 (p=0.03013) (S)

Table 2 shows that the maximum patients (81.02%) suffered from moderate depressive episodes (F 32.1), followed by severe depressive episodes (8.75%) (F 32.2), organic depressive disorders (7.29%) and a minimum with mild depressive episodes (2.91%). The association between age and depressive disorders was significant, showing that the maximum number of patients in all age groups had moderate depression.

**Table 2:** Distribution of patients with depressive disorders

Depressive disorders	Age group (years)				%
		
	65-69	70-74	75-79	>80	
Mild (F 32.0)	0	4	0	0	2.91
Moderate (F 32.1)	49	45	8	9	81.02
Severe (F 32.2)	5	4	1	2	8.75
Organic (F 06.32)	2	4	4	0	7.92

Total	56	57	13	11	100

Chi-square=19.56, df=9, p<0.05 (p=0.0208) (S)

Table 3 shows that the maximum co-morbidity was found in neurological cases (70%), followed by endocrinal cases (59.25%) and the least among patients who had a surgical diagnosis (22%). The extent of depression was maximum (60%) among neurological conditions followed by endocrinal cases (59.37%), cardiovascular cases (56.86%), and minimum in gynaecological cases. Adjustment disorders were maximum among surgical inpatients (55%) followed by inpatients suffering from injuries (47.36%). Anxiety disorders were more commonly seen among gynaecological inpatients followed by patients with infectious diseases. The maximum number of cases with dementia were (15%) among those with neurological conditions. The maximum number of patients with delirium were found among those with endocrinal and metabolic conditions (15.62%) followed by neurological conditions (15%).

**Table 3:** Medical-surgical diagnosis versus psychiatric co-morbidity

					Disorders						
					
Medical-surgical illnesses	Medical-surgical diagnosis	Total	With psychiatric illness (%)	No psychiatric illness	Depression	Adjustment	Anxiety	Dementia	Delirium	Bipolar	Substance-related disorders
I	Infections	103	55 (53.40)	48	29	11	11	2	2	0	0
II	Injuries	38	19 (50)	19	8	9	0	1	1	0	0
III	Endocrinal	54	32 (59.25)	22	19	4	1	3	5	0	0
IV	Carcinoma	37	21 (56.75)	16	12	7	0	1	1	0	0
V	Surgical	91	20 (21.97)	71	7	11	0	2	0	0	0
VI	Gynaecological	9	6 (66.66)	3	1	2	2	1	0	0	0
VII	Cardiovascular	109	51 (46)	58	29	9	6	2	1	2	2
VIII	Neurological	57	40 (70)	17	24	0	2	6	6	2	0
IX	Others	30	16 (53.33)	14	8	5	2	1	0	0	0

Total	528	260	268	137	58	24	19	16	4	2	0

Computed Chi-square at 8 degrees of freedom = 44.13 (**)

Note: Critical Chi-square values at 5% and 1% levels are 15.50 and 20.09, respectively

## DISCUSSION

The rate of psychiatric co-morbidity in the present study was 49%. It is in agreement with the findings of Uwakwe^20^ and Bowler *et al.*^17^ who found psychiatric co-morbidity up to the extent of 45.3% and 47.1%, respectively. These findings differ from the study done by Muller *et al.*^16^ in Germany, who reported a rate of 62% of psychiatric co-morbidity among elderly inpatients. These wide variations are because of differences in the social and geographical backgrounds. However, Schuckit *et al.*^6^ revealed that 24% of medical–surgical illlnesses over the age of 65 years at the La Jolla Veterans Administration Hospital had unrecognized major mental disorders. The study by Schuckit *et al.*^6^ was different from the present study because of sample characteristics, as this study was conducted among veteran inpatients.

Dube^31^ and Premarajan^13^ carried out their respective studies in urban communities and found psychiatric morbidity of the order of 22.34% and 17.3%, respectively. Nandi *et al.*^10^ and Tiwari *et al.*^11^ observed a prevalence of psychiatric disorders in rural communities to be as high as 33% and 42.2%, respectively. Different rates of psychiatric disorders reported in these studies may be because some of them targeted the urban population while others concentrated on the rural population. But all are unanimous in reporting the higher prevalence of psychiatric disorders in the elderly population. When we compare these field-based studies with the present study, which shows a higher psychiatric co-morbidity, we conclude that medical–surgical illness in the geriatric population increases the psychiatric co-morbidity, thus emphasizing that special attention is needed for this ever-increasing group.

Avasthi *et al.*^14^ reported the distribution of psychiatric disorders in 107 referred geriatric inpatients to be 8.6%; 48 cases of organic psychosis, 26 neurosis, 19 other disorders and 14 no psychiatric disorder. The variation in results as compared to the present study may be because of the fact that in our study we screened geriatric inpatients for psychiatric illness while in their study the patients were referred by clinicians who suspected psychiatric illness.

In the sample under study, males outnumbered females but the rate of psychiatric co-morbidity was more in females as compared with males. This is in conformity with earlier field- based studies of the elderly population in India.^9^,^10^,^12^,^13^ Depression, anxiety and adjustment disorders were more common in females as compared with males. These results are contrary to the study carried out by Muller *et al.*^16^ in Germany, in which men were more frequently affected than women.

In terms of the diagnostic break-up, depression was the most common psychiatric disorder in this study (25.94% of the total study participants). In psychiatrically ill patients 56% are depressed. A majority of inpatients had moderate depression (81.02%). Tiwari *et al.*^11^ found depression up to the extent of 30.09% in a psychiatrically ill geriatric rural population. Ramchandran *et al.*^7^ estimated the prevalence of mental disorders in those aged 50 years and above and observed that 24.1% had depression. Banerjee and MacDonald^32^ in their Lewrisham study found depression in 26% of their sample comprising persons 65 years of age and above. Uwakwe^20^ in his hospital-based sample observed that depression was the most common co-morbidity. Muller *et al.*^16^ reported on psychiatric morbidity in elderly inpatients and found depressive disorders in 22%.

It was observed during this study that not a single patient was receiving treatment for depression. However, depression in medically ill patients can slow recovery, exacerbate medical illness, can be misdiagnosed as organicity resulting in longer hospitalization, and may even contribute to mortality. Therefore, it is suggested that physicians should maintain a high index of suspicion for depression in patients with significant medical illness and aggressively treat the condition when indicated.

Dementia was found in 3.6% of patients in this study. However, it was observed that the primary physician was unaware of the presence of dementia. Depression may also be present early in the course of dementia and it may mimic subacute delirium, thereby complicating the diagnosis.

The prevalence of dementia in the Indian population differs greatly from the western population. Muller *et al.*^16^ and Bowler *et al.*^17^ reported the prevalence of dementia in elderly medical/surgical inpatients to be very high, i.e. 26% and 26.8%, respectively. In India, the prevalence of dementia as such was low as reported by earlier field surveys. Nandi *et al.*^10^, Rao and Madhavan,^8^ Ramchandran *et al.,*^7^ Shaji *et al.*^9^ reported the prevalence of dementia to be 1.6%, 0.88%, 3.2% and 3.39%, respectively. This fact is also corroborated by White^19^ who reviewed cross-cultural research on the epidemiology of dementia and came to the conclusion that estimated rates of dementia were somewhat lower in Asian countries than those reported from Europe and the USA.

In the present study, delirium was found in 3% and most of the patients (1.13%) were suffering from neurological conditions, closely followed by endocrinological conditions (0.94%). However, Bowler *et al.*^17^ reported a very high rate of delirium, i.e. 11.1% as they carried out their study in an acute medical unit for the elderly.

## CONCLUSION

A medical setting is an important *de facto* mental health setting in which the mental sufferings of patients could be ameliorated. This would not only help in the successful management of patients but would also allow cost-effective planning of health services. This has led to the concept of consultation–liaison psychiatry, where the psychiatrist becomes an important part of the medical–surgical team and psychological morbidity is treated at an early stage. For providing better health services to geriatric inpatients, a coordinated approach of the geriatric and psychogeriatric services is needed. Thus, the role of consultation– liaison psychiatry comes into play.

### Limitations

- Patients from the eye department could not be interviewed because of non-availability as they were not advised overnight admission.
- All patients were screened in a one-stage interview irrespective of their stay in the hospital. Uncooperative patients were not included as they or their informants were not ready for interview.
- This hospital-based sample did not represent any particular geographical area as the study was undertaken in a tertiary care hospital.
- The effect of treatment on outcome has not been evaluated.

### Strengths

This study is the first of its kind to stress on the psychological needs of geriatric inpatients and on consultation-liaison psychiatry.

### Future direction

Based on this study, further research should be carried out in India to check the cost-effectiveness of consultation–liaison psychiatry and efforts should be made to give consultation– liason psychiatry a practical shape^33^.
